# Multimodal imaging in a pedigree of X-linked Retinoschisis with a novel *RS1* variant

**DOI:** 10.1186/s12881-018-0712-8

**Published:** 2018-11-12

**Authors:** Kirk Stephenson, Adrian Dockery, Niamh Wynne, Matthew Carrigan, Paul Kenna, G. Jane Farrar, David Keegan

**Affiliations:** 1 The Catherine McAuley Centre, Mater Private Hospital, Nelson Street, Dublin 7, Ireland; 2 Department of Genetics, Trinity College Dublin, Dublin, Ireland; 3 The Research Foundation, The Royal Victoria Eye and Ear Hospital, Dublin, Ireland

**Keywords:** X-linked Retinoschisis, Retinoschisin, Inherited retinal dystrophy, Inherited maculopathy

## Abstract

**Background:**

To describe the clinical phenotype and genetic cause underlying the disease pathology in a pedigree (affected *n* = 9) with X-linked retinoschisis (XLRS1) due to a novel *RS1* mutation and to assess suitability for novel therapies using multimodal imaging.

**Methods:**

The Irish National Registry for Inherited Retinal Degenerations (Target 5000) is a program including clinical history and examination with multimodal retinal imaging, electrophysiology, visual field testing and genetic analysis. Nine affected patients were identified across 3 generations of an XLRS1 pedigree. DNA sequencing was performed for each patient, one carrier female and one unaffected relative. Pedigree mapping revealed a further 4 affected males.

**Results:**

All affected patients had a history of reduced visual acuity and dyschromatopsia; however, the severity of phenotype varied widely between the nine affected subjects. The stage of disease was classified as previously described. Phenotypic severity was not linearly correlated with age. A novel *RS1* (Xp22.2) mutation was detected (NM_000330: c.413C > A) resulting in a p.Thr138Asn substitution. Protein modelling demonstrated a change in higher order protein folding that is likely pathogenic.

**Conclusions:**

This family has a novel gene mutation in *RS1* with clinical evidence of XLRS1. A proportion of the older generation has developed end-stage macular atrophy; however, the severity is variable. Confirmation of genotype in the affected grandsons of this pedigree in principle may enable them to avail of upcoming gene therapies, provided there is anatomical evidence (from multimodal imaging) of potentially reversible early stage disease.

## Background

Target 5000 [1, 2] (Fighting Blindness, Ireland), the Irish National Inherited Retinal Degeneration Registry, aims to phenotype and genotype all patients in Ireland with inherited retinal degenerations (IRD). A concurrent goal of the study is to facilitate the implementation of individualised management plans including, where appropriate, novel therapeutic options for those patients with modifiable disease. This paper is focused on the application of this process for a pedigree with X-Linked Retinoschisis (OMIM: 312700, XLRS1) and the clinical and genetic workup of these patients for potential new therapies and future participation in appropriate trials now emerging for this form of IRD.

XLRS1 is a rare IRD (1:15000–30,000 [3]) due to variants in the *RS1* gene encoding the retinoschisin protein (OMIM: 300839, Xp22.1). XLRS1 is a mutationally heterogeneous disorder with over 230 known variants [4–6], the majority of mutations falling in exons 4–6 (the structurally important discoidin domain of the retinoschisin protein [7]). Congenital poor vision in males is due to macular schisis and results in variable outer retinal atrophy with age. As novel gene therapies are in clinical trials [8, 9], to optimise options for patients, confirmation of the presence of an *RS1* variant is essential and moreover must be supplemented with various clinical assays to assess the stage of disease and potential suitability for treatment.

With wide variability in phenotype between and within families [10–12], clinical diagnosis can be challenging. Multimodal imaging has been an excellent addition for confirming phenotype and guiding molecular genetic testing. Optical Coherence Tomography (OCT) imaging may detect intraretinal cystic spaces [13], which may appear in multiple retinal planes. Fundus autofluorescence (AF) imaging can detect subtle patterns of change in the retinal pigment epithelium (RPE), which allows staging of severity [14]. Microperimetry can delineate areas of residual function [15]. Thus, multimodal imaging can be used to demonstrate maintenance of anatomy which may determine suitability for novel treatments.

The *RS1* genomic location was first implicated as pathogenic using multipoint linkage analysis of Dutch families affected by XLRS1 [16]. Locus localization was later refined and the *RS1* gene was identified by positional cloning. Mapping and expression analysis led to the discovery of the then novel transcript (XLRS1) [17] encoding the retinoschisin protein.

Retinoschisin, a 224 amino acid protein, is secreted from photoreceptors and bipolar cells in retina and has been shown to be crucial for cell adhesion during retinal development [18]. It has been suggested that retinoschisin may also be involved in mitogen-activated protein kinase signaling and apoptosis in retina [18] and recently has been shown to influence Na/K-ATPase signaling and localization [19]. Employing cryo-electron microscopy, it has been discovered that functional retinoschisin forms a dimer of octamer rings comprising a hexadecamer. Given this intricate structure, a mutation that affects the folded structure of the RS1 protein monomer is likely to inhibit the proper assembly of the 16-subunit oligomer [20, 21] as evidenced by the large number of reported pathogenic mutations [5]. Pathogenic RS1 mutations are typically associated with XLRS1, although some mutations show greater phenotypic variability than others, including greater ranges of onset age and severity of condition [12].

## Methods

### Clinical phenotyping

**Fig. 1:**
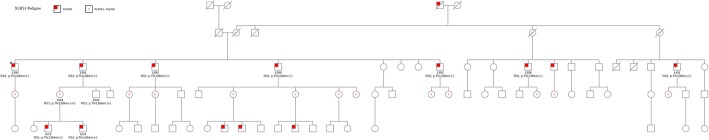
Pedigree of 5 generations of an XLRS1 pedigree. Individuals marked with a red square were confirmed to be clinically affected. *RS1* genotype has been annotated within the pedigree tree for those investigated

A large pedigree with a clinical diagnosis of XLRS1 was invited to participate given informed consent. Nine affected males (7 adults, 2 children), one carrier female and one unaffected male attended the recruiting hospitals as specified above to take part in the IRD registry (Fig. 1).

Phenotyping included an ophthalmic and medical history, pedigree mapping, and dilated ophthalmic examination. Colour photography, autofluorescence (AF, Optos plc, Scotland) and spectral domain optical coherence tomography (SD-OCT, Cirrus HD-OCT, Carl Zeiss Meditec AG, CA, USA) images were acquired. This multimodal imaging was assessed and the macular findings were categorised into their appropriate stages. Stage was assessed by individual eye, not entire patient.

### DNA isolation and next generation sequencing

Blood samples were taken from patients after clinical assessment. DNA was isolated from 2 ml of blood and fragmented for targeted sequencing to an average fragment size of 200–250 base pairs. Sequencing libraries were generated and target capture was performed with the Nimblegen SeqCap EZ kit (Roche), incorporating the exonic regions of over 200 genes implicated in IRDs. Capture regions also included intronic regions in the CEP290, ABCA4 and USH2A genes that are known to potentially contain pathogenic mutations [22–24]. The total size of the captured region was approximately 750 kb.

Captured patient DNA was multiplexed into 24-sample pools and sequenced using an Illumina MiSeq. Confirmatory single-read sequencing was also performed to verify the presence of candidate mutations.

### Polymerase chain reaction and sanger sequencing

To validate the *RS1* variant identified by NGS, an amplicon for direct Sanger sequencing was designed incorporating the variant. The sequence used for primer design was Human reference transcript NM_000330.3. Forward primer: 5′- GCAGATGATCCACTGTGCTG – 3′. Reverse primer: 5′ - TTTCTTGGGAGGTGGAGATG – 3′. Oligonucleotides were purchased from Sigma-Aldrich (www.sigmaaldrich.com/). The target DNA products were amplified using Q5 High-Fidelity 2x Master Mix (New England Biolabs Inc). The annealing temperature for reactions was 65 °C; all other details were executed as per the supplier’s recommendations. PCR products were purified using the GeneJET Gel Extraction Kit (Thermo Fisher Scientific). Sanger sequencing was performed by Eurofins Genomics (www.eurofinsgenomics.eu).

### Data analysis

Data obtained from NGS was subsequently demultiplexed and mapped to the human genome (hg38) using BWA version 0.7.15 [25]. Duplicate reads were flagged using Picard version 2.5.0 [26] and downstream analysis and variant calling performed using Freebayes version 1.1.0 [27].

Variants were identified and scored based on methods outlined in Carrigan et al. [1]. Synonymous variants, polymorphisms and mutations with high frequency in any population were filtered out, and the remaining list of rare variants with the potential to affect protein sequence was output for manual inspection. Output scores from the following ensemble variant pathogenicity predictor tools are shown in Table 1. The scale for each score ranges from 0 (likely benign) to 1 (likely pathogenic).

**Table 1:** A direct comparison of the novel mutation, p.Thr138Asn, and a known pathogenic variant in *RS1*. Both variants were analysed with the same bioinformatic pipeline and have been scored by the same computational tools

Variant	Nucleotide	Protein	DBSNP ID	MetaLR	M-CAP	REVEL	ClinVar Report
Novel	c.413C > A	p.Thr138Asn	None	0.9742	0.8775	0.929	None
Known	c.636G > A	p.Arg209His	rs281865362	0.9446	0.9233	0.796	Pathogenic

#### MetaLR

Meta Logistic Regression (LR) [28] incorporates pathogenicity prediction scores and maximum minor allele frequency from nine different tools for more accurate and thorough evaluation of deleterious effect of missense mutations. This allows for the more accurate assessment of variants than any of the singular methods alone.

#### M-CAP

Mendelian Clinically Applicable Pathogenicity (M-CAP) Score [29] was the first high sensitivity pathogenicity classifier for rare missense variants in the human genome aimed at the clinic. It combines pathogenicity scores from other tools and databases (including SIFT, Polyphen-2 and CADD) with novel features to create a more powerful model.

#### Revel

REVEL (rare exome variant ensemble learner) [30], is also an ensemble predictor tool and was trained with recently discovered pathogenic and rare benign missense variants, excluding those previously used to train its constituent tools. REVEL also claims to have the best performance for categorising pathogenic from rare benign variants with allele frequencies < 0.5%.

The American College of Medical Genetics and Genomics (ACMG) criteria for classifying pathogenic variants was utilised [31]. The other rare variants detected were for autosomal recessive conditions and showed pathogenic variants in only 1 allele. This was consistent between affected male relatives in this study.

### Protein modelling

3D models of single subunit wildtype RS1 (NM_000330.3) and the Thr138Asn mutant were generated using Iterative Threading ASSEmbly Refinement, I-TASSER [32]. Polymer structures of RS1 were obtained from the Protein Data Bank (PDB, ID#3JD6,) [33]. The effect of single point mutations on protein stability was measured using STRUM [34]. Protein alignments were generated using Clustal Omega [35].

## Results

Mean patient age of affected adult males (*n* = 7) was 67 +/− 5.38 years. The recruited carrier female (daughter) was 47 years of age. Mean age of the 2 affected grandsons was 13 +/− 1.41 years. Mean visual acuity for adult affected males was LogMAR 0.78 +/− 0.16 for right eyes and 0.74 +/− 0.28 for left eyes. Two eyes were not assessable due to one case of dense cataract (amblyopic eye) and one enucleation (due to complications following previous retinal surgery, presumed post vitreous haemorrhage or detachment). Only one of 14 adult eyes developed either a retinal detachment or haemorrhage previously (indeterminable). Mean visual acuity of affected grandsons was LogMAR 0.6 +/− 0.14 for right eyes and 0.5 for both left eyes.

**Fig. 2:**
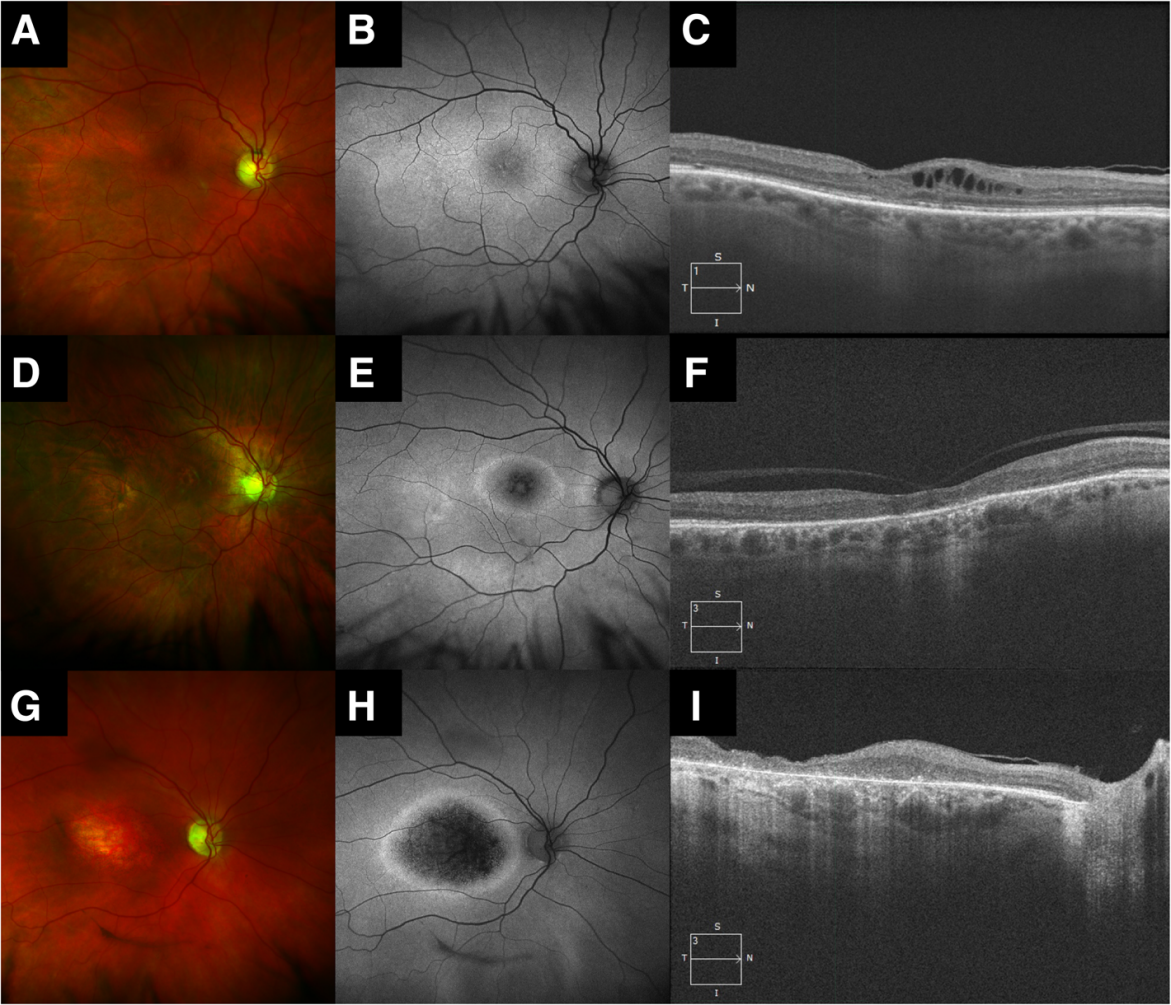
Phenotypic features of selected cases from this XLRS1 pedigree. Colour photographs (left column), autofluorescence (middle column), and SD-OCT (right column) of the right eyes of 3 patients in Stage 1 (top row), Stage 2 (middle row) and Stage 3 (bottom row). Further description is available in Table 2 and the ‘Results’ section

**Fig. 3:**
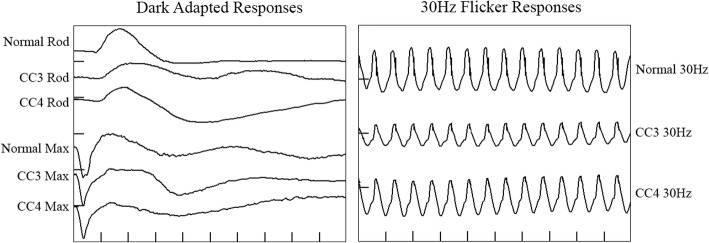
ISCEV full-field electroretinogram [45] responses from affected grandsons (CC3 and CC4). Horizontal axis: 50 msec per division; Vertical axis: Dark Adapted Responses 500 μV per division; 30 Hz Flicker Responses 250 μV per division. The rod-isolated responses of CC3 and CC4 were slightly, but significantly reduced in amplitudes. The mixed rod and cone responses to the maximal intensity flash stimulus presented to the dark-adapted eye showed a normal a-wave amplitude in each subject, (CC3 445 μV, CC4 441 μV). The b-wave amplitudes were significantly reduced in each patient (CC3 515 μV, CC4 502 μV) and approximated the a-wave amplitudes. Ordinarily, the b-wave amplitude greatly exceeds that of the a-wave. The normal a-wave and selective reduction of the b-wave indicates preserved photo-transduction but impaired post-receptoral retinal function. This has been reported in RS1-associated X-linked retinoschisis [44]

Macular changes varied in severity, which showed no correlation to patient age. A severity staging was proposed by Tsui and Tsang [14], outlined in Table 2. Of 12 assessable adult eyes, 1 was in Stage 1 (8%, mean age 74y), 6 were in Stage 2 (50%, mean age 65.6 +/− 5.72y) and 5 were in Stage 3 (42%, mean age 68.8 +/− 4.55y). The predominantly advanced stage of disease here is likely due to advanced patient age. Corresponding images from this series are in accordance with this and are shown in Fig. 2. Both affected grandsons had bilateral Stage 1 disease. Electrophysiology data for the 2 affected grandsons is shown in Fig. 3. Confirmation of this *RS1* variant’s pathogenicity will be of most benefit for this younger generation with potentially reversible macular changes.

**Table 2:** The clinical staging of Retinoschisis [14]

Stage	Colour	AF	SD-OCT
1	Macular schisis, classically radial (A)	Macular pigments displaced between schitic areas (B)	≥1 intraretinal schisis plane (C)
2	RPE pigment mottling (D)	Hyperautofluorescent ring at macula (E)	Collapse of schisis plane(s) (F)
3	Macular atrophy (G)	Large area of central hypoautofluorescence with surrounding hyperautofluorescent ring (H)	Outer retinal atrophy (I)

Figure 2 demonstrates the clinical staging of XLRS using multimodal imaging. Patient 1 (74y) demonstrates the features of Stage 1 (mild) disease with BCVA LogMAR 0.6. There are subtle pigmentary changes on colour photography (A), with displacement of macular pigment leading to radial pattern of hyper- vs hypo-autofluorescence (B). The most striking feature is on OCT with intraretinal cystic spaces (C) in the inner plexiform and inner nuclear layers.

Patient 2 (60y) shows Stage 2 (intermediate) disease with BCVA of LogMAR 0.9. Colour photography reveals a hypopigmented ring at the macula, surrounding an area of normal pigmentation (D). Autofluorescence confirms this as hypoautofluorescent with a surrounding ring of hyperautofluorescence and a preserved iso-autofluorescent foveal area (E). OCT reveals the absence/collapse of intraretinal cystic spaces, but lacking complete outer retinal atrophy (F). Incidental vitreo-foveal traction is noted in this case.

Patient 3 (71y) has stage 3 (advanced) disease with BCVA of LogMAR 1.0. The colour photograph demonstrates macular atrophy (G), which appears as hypoautofluorescence with a hyperautofluorescent ring (H). This corresponds to outer retinal atrophy without schitic intraretinal space on OCT (I).

Next generation sequencing detected a novel missense mutation in *RS1* (c.413C > A; p.Thr138Asn [1]) falling within exon 5 of the discoidin domain [7]. This is a variant of unknown significance; however, due to its location adjacent to known pathogenic loci, it was deemed to be potentially pathogenic and in silico analysis was undertaken.

### Mutational analysis

**Fig. 4:**
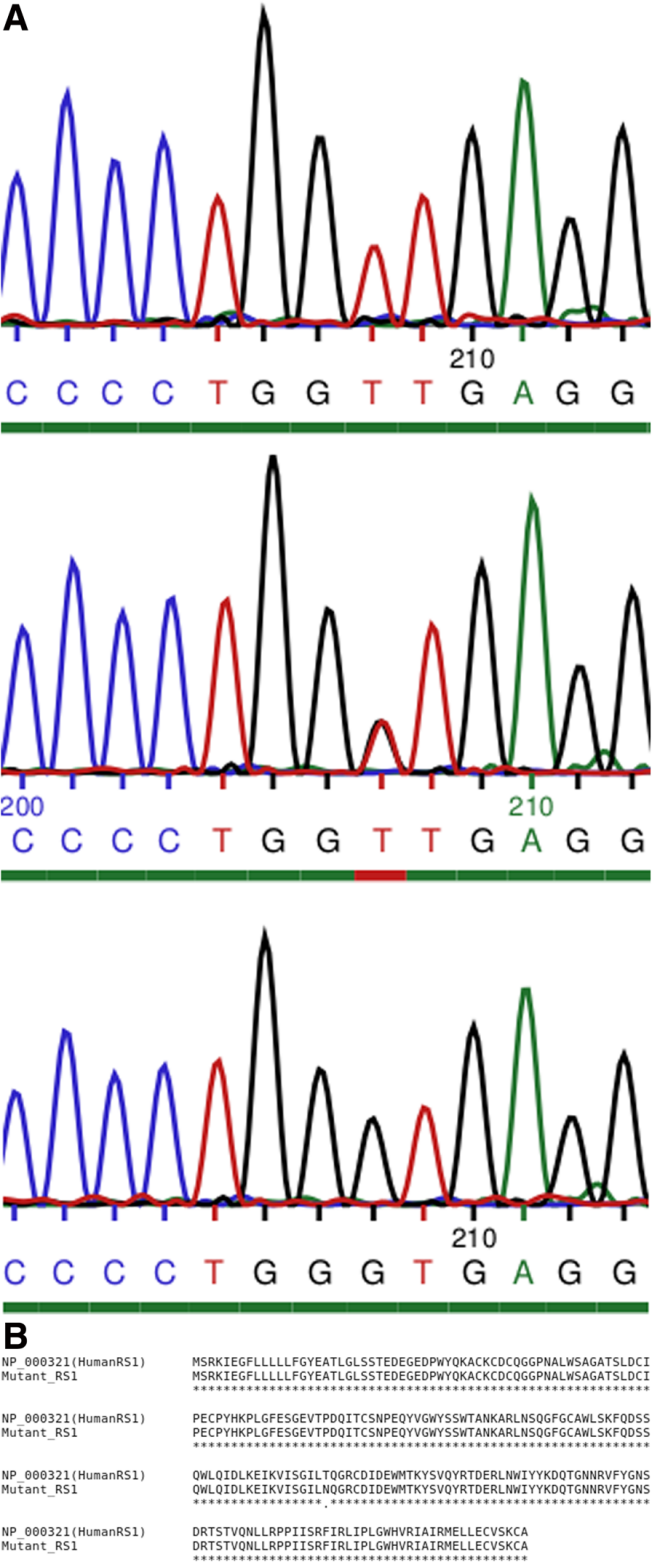
**a** Sanger sequencing of *RS1*-exon 5 polymerase chain reaction (PCR) products from members of the pedigree. Top: Sanger sequencing result for affected male (ID#1392) showing a T nucleotide trace at sequencing position 208. Middle: Sanger sequencing result for carrier female (ID#1534) showing a heterozygous result at sequencing position 207, revealing traces from both signals of wild-type G allele and the mutant T allele. Bottom: Sanger sequencing result for unaffected male (ID#1634) showing a G nucleotide trace at sequencing trace position 208. **b** Clustal Omega results for protein alignment of reference RS1 protein sequence against the observed mutant protein sequence. “.” denotes the position of the substitution

The exonic regions from a panel of 218 IRD genes were sequenced by NGS in the proband from this pedigree. An additional 5 affected males were sequenced with a subsequent panel of the exonic regions of 254 IRD genes. Analysis of this NGS data led to the identification of a mutation in the *RS1* gene within the pedigree. Further analysis of each of the members of the family by PCR amplification of *RS1* and Sanger sequencing demonstrated that each affected male was found to have a hemizygous nonsynonymous mutation in exon 5 of the *RS1* gene (NM_000330.3:c.413C > A, p.Thr138Asn). In addition, the single carrier female analyzed was heterozygous for the same *RS1* mutation while the unaffected male was hemizygous for the reference base at that position (Fig. 4a, b).

**Fig. 5:**
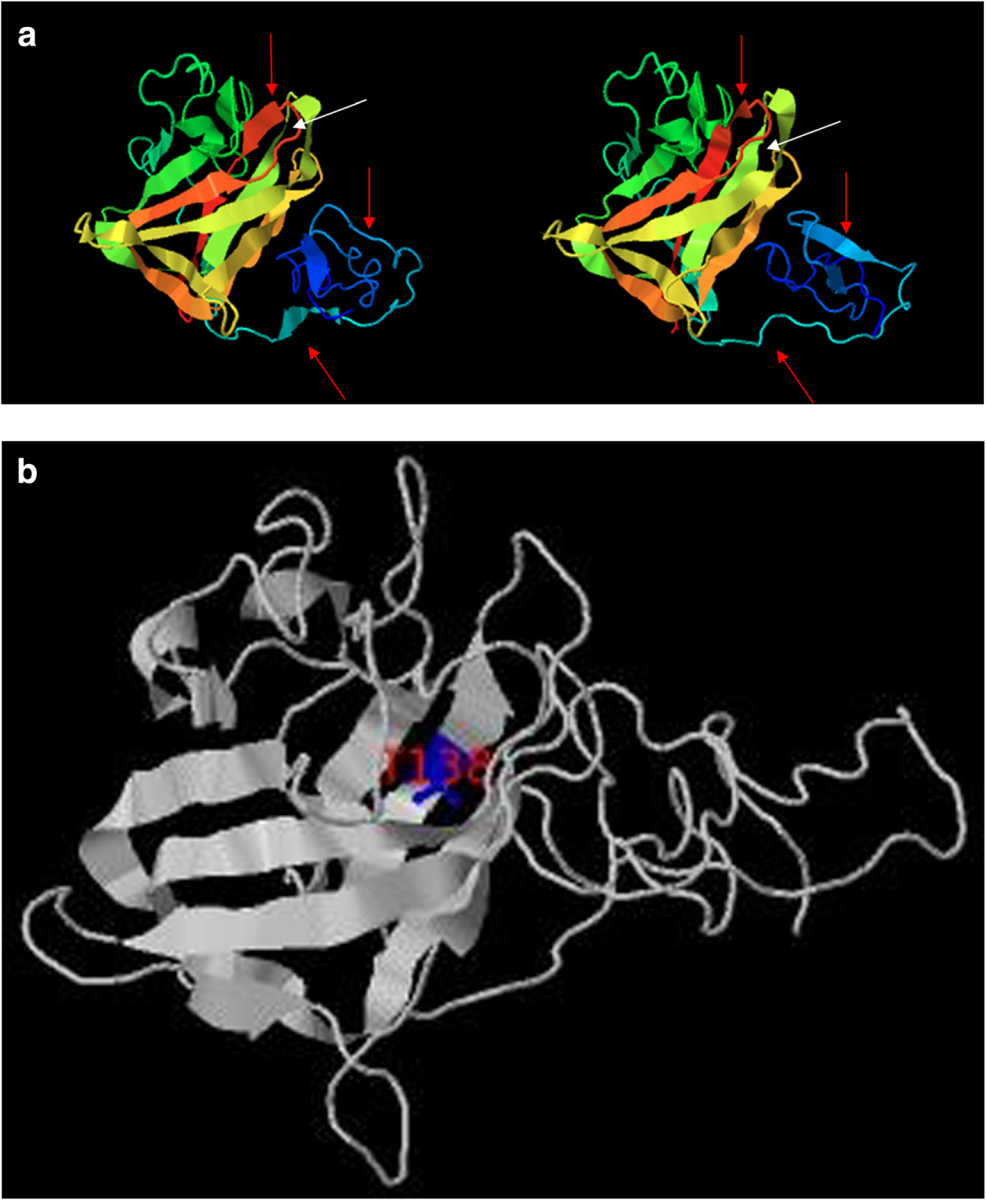
**a** Protein models of reference RS1 (left) and mutant T138 N (right) proteins generated by the I-TASSER program. Red arrows denote obvious structural changes to protein structures. A white arrow highlights the position of the amino acid change at codon 138. **b** Protein model of RS1 mutant T138 N generated by STRUM. The affected amino acid residue is displayed in blue and annotated in red

**Fig. 6:**
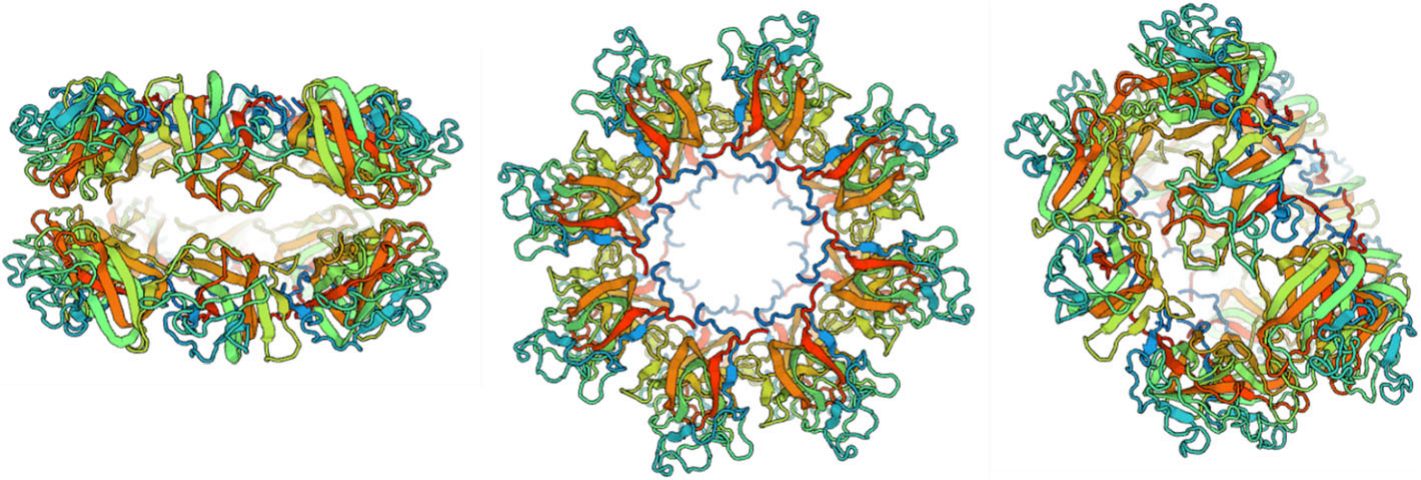
Different views of the back-to-back octamer rings of wild type RS1 obtained from the Protein Data Bank. (Left) Objective view. (Middle) Plan view. (Right) Perspective view

The p.Thr138Asn *RS1* mutation involves a polar to polar amino acid substitution. Given that this novel as yet previously unreported mutation is located in the critical discoidin domain of the retinoschisin protein, where many pathogenic mutations have been identified previously, additional in silico analysis was undertaken to explore the potential pathogenicity of this *RS1* mutation on protein structure and conformation. The destabilizing effect of this mutation as predicted in silico is shown below in terms of a prediction of the changes to fold stability (Table 3), where a negative delta delta G (ddG) score is indicative of a destabilizing effect. This change is also represented visually as theoretical structural changes to the retinoschisin monomeric protein structure (Fig. 5a, b). Notable secondary structural changes are identified with arrows in Fig. 5a while Fig. 5b clearly visualizes the position of the specific amino acid substitution. The complexity of the hexadecameric structure of retinoschisin is shown in Fig. 6 for reference.

**Table 3:** Output results of the STRUM program. The change in stability scores between the mutant and wild type RS1 protein is given as delta delta G (ddG)

Position	Wild Type	Mutant Type	ddG
138	T	N	−0.78

Table 1 displays the comparative output scores of three highly regarded variant pathogenicity prediction tools for this novel variant and a known pathogenic variant. As seen in the respective columns of the tools, both variants have been deemed likely pathogenic by all three tools. Also, the novel variant described in this study is scored more likely to be pathogenic by two out of three methods listed.

The ACMG guidelines [31] have also been implemented to classify the novel variant. The relevant information has been outlined in Table 4 along with supporting evidence where appropriate [20, 36, 37]. The guidelines state that there is sufficient evidence to classify this variant as pathogenic as there are one strong (PS1) and three moderate (PM1, PM2 and PM5) lines of evidence.

**Table 4:** The ACMG guidelines applicable to the novel variant in question, p. Thr128Asn

Category Code	Qualifying Criteria	Relevance to Case
PS4	The prevalence of the variant in affected individuals is significantly increased compared to the prevalence in controls.	This variant is found in a sufficient number of relatives to qualify it as strong evidence of pathogenicity [36].
PM1	Located in a mutational hot spot and/or critical and well-established functional domain (e.g. active site of an enzyme) without benign variation.	Located in well established functional domain as reported by several studies [20, 37].
PM2	Absent from controls (or at extremely low frequency if recessive) in Exome Sequencing Project, 1000 Genomes or ExAC.	Absent from every databased queried.
PM5	Novel missense change at an amino acid residue where a different missense change determined to be pathogenic has been seen before.	Novel mutation at same amino acid as previously reported p.Thr138Ala (HGMD Database).
PP1	Co-segregation with disease in multiple affected family members in a gene definitively known to cause the disease.	Cosegregation in a family in a gene known to cause disease.
PP3	Multiple lines of computational evidence support a deleterious effect on the gene or gene product.	Multiple lines of computational evidence presented here (Table 1).
PP4	Patient’s phenotype or family history is highly specific for a disease with a single genetic etiology.	Patient phenotype is highly specific for a disease.

## Discussion

A key function of retinoschisin is in retinal adhesion [7]. There is some debate over which retinal plane is affected by the schisis, which has been documented on OCT at the level of the photoreceptor inner segments, the inner nuclear layer and the nerve fibre layer [13, 38]. Decreased retinoschisin protein function may, and likely does, cause multiple planes of schisis.

There is significant variability of the severity of disease phenotype between the two eyes of the same individual, between different members of this XLRS1 pedigree and, as previously documented, between unrelated individuals [10–12]. This variability appears greater with age with some patients progressing through the clinical stages at different rates, possibly due to acquired/environmental factors or other genetic modifiers (see below). Although this variability is noted, there are clinical characteristics that guide the clinician to this diagnosis (e.g. pedigree, peripheral retinoschisis, lack of subretinal flecks or vitelliform changes) which are supported by a likely-pathogenic *RS1* variant and lack of other known pathogenic genetic variants. This supports the bimodal presentation of XLRS1. Severe cases of the disease (approximately 30%) are detected in infancy with poor fixation and/or strabismus. Milder cases (approximately 70%) are detected at school entry or in adulthood [39]. The current study highlights that the underlying genetic variant on the *RS1* gene is not predictive of phenotypic severity for this, or indeed any of the other, genetic variant(s) that have been described [12]. Thus, in assessing patients for trial/intervention suitability both a genetic and full phenotypic analysis is required. For example, gene therapy will not be beneficial if there is significant structural change (i.e. stage 3). However, the combination of a stage 1 or 2 case with confirmed genotype would suggest a possible role for genetic manipulation/replacement. As our knowledge of the impact of multiple variants increases we may better predict the molecular effects of each on retinal structure and function thus tailoring our approach to intervention further.

The clinical findings may be subtle in early stage or mild forms of disease. Classically, radial cystic maculopathy at the fovea is seen in 98–100% of cases [40] (Fig 2a-c). Ancillary tests have proven useful, particularly in subtle cases. An electronegative electroretinogram is suggestive of, but not specific, to XLRS1 [41] as this feature may be seen in acquired disease of the inner retina (e.g. central retinal artery occlusion). Microperimetry may assist in detection of subclinical disease [15]. Late stage disease is a diagnostic challenge; however, clinical examination, family history and genotyping as outlined above will aid in determining the diagnosis.

Colour photography, Autofluorescence and Optical Coherence Tomography are non-invasive and accessible tests useful in determining the clinical stage of XLRS1. This is relevant, for current and upcoming gene therapy trials [8, 9], in selecting those cases most likely to benefit from intervention (i.e. early to intermediate disease [40]). Ancillary tests, such as adaptive optics (AO) imaging and microperimetry, can complement assessment of cone structure/spacing in those who are deemed early/intermediate stage [42, 43].

To our knowledge, this specific mutation is currently unreported in any clinical database available including the Leiden University’s Open Variation Database for Retinoschisis (LOVD 3.0 [6]) and represents a novel and likely pathogenic mutation given the clear segregation in this pedigree and the significant predicted effects on protein structure.

Recent studies have outlined the importance of certain key residues in correctly assembling a functional RS1 oligomer. Several key residue positions necessary to ensure proper folding of the retinoschisin protein have been identified [20]. Of note, amino acids at positions 137 and 139 were highlighted in that study as fundamental to forming a beta-sheet required in the interaction between subunits essential in the formation of their functional octamer ring structure. It is possible therefore, that an amino acid substitution at position 138 may provide some steric hindrance, that in turn, would interfere with the relationship between different subunits on the same octamer ring. Such interference could in principle have deleterious consequences for the formation and stability of the oligomeric RS1 complex. This hypothesis is further supported by in silico analysis of the p.Thr138Asn mutation. Computational analysis was utilized to predict protein folding changes in the mutant protein (using I-TASSER); physiochemical properties, position specific conservation and secondary structure prediction scores were used to determine the destabilization effect(s) of the protein substitution at position 138 (using STRUM) where significant changes in the above were predicted for the p.Thr138Asn mutation.

The prediction scores in Table 1 are useful for assessing the confidence with which a variant can be called deleterious or not. However, these are confidence scores and not pathogenicity scores, so although the scores listed would be considered to be quite high, these scores cannot indicate the detrimental effect that this mutation may have relative to a similar amino acid substitution elsewhere in the protein. Increased bioavailability of functional retinoschisin protein would logically correlate to the lasting preservation of the retinal layers; however, to assess this dosage effect, each variant would have to be assessed individually for function and half-life. This is a challenging task for retinoschisin as it has typically proven quite difficult to produce in useful quantities [20]. Currently we rely on in silico predictors of a variant’s effect on protein structure. This includes protein modelling (Figs. 4a and b) where possible and delta delta g scores (Table 3), to predict the destabilising effects a mutation may have on a protein. However, when all lines of evidence are considered, this novel *RS1* variant is classified as pathogenic under the guidelines outlined by the ACMG [31] (Table 4).

In XLRS1, age is not an absolute factor, particularly in adulthood, when evaluating severity of disease and the likely benefit from novel gene therapies. The individuals in this XLRS1 pedigree exhibited no clear correlation between patient age and 1) stage of disease, 2) visual acuity, 3) central retinal thickness or 4) age at onset despite a shared novel *RS1* gene variant. Advanced cases prove a diagnostic challenge as many inherited and acquired macular diseases follow a final common pathway of outer retinal atrophy. Each case must be judged on its potential (anatomically within Stage 1 or 2 and genetically possessing a pathogenic *RS1* variant) to respond to novel therapies. Furthermore, the variability in clinical presentations between family members with the same mutation suggests the possible involvement of genetic modifier loci and or environmental effect(s).

While there is variability in phenotype between relatives affected by the same genetic variant, the significance is that the clinician not be discouraged from the diagnosis due to these differences but consider them within the spectrum of the 3 described clinical stages. The diagnostic value of ERG, in particular the characteristic decrease in b-wave activity in combination with suggestive clinical findings are powerful tools for diagnosing younger patients, however the diagnostic value of these tests may be less certain with advanced age [44]. Confirmation of extent of severity (clinical stages) and a pathogenic *RS1* variant open a potential avenue of treatment for those in Stage 1 or 2. This information then guides genetic testing for at risk relatives who could possibility benefit from novel disease-modifying therapies.

There is a possibility that there may be some form of modifier or digenic effect which could account for the observed phenotypic variability. With this in mind, the coding regions of known genes associated with retinal degenerations were subjected to sequence analysis. The presence of multiple pathogenic variants within a single patient is extremely rare but has been previously observed, even in our own IRD cohort. Although, as previously mentioned, this method unfortunately did not detect any likely genetic cause of phenotypic variability between the affected patients in this pedigree.

The retinoschisin protein is widely expressed throughout the layers of the retina in childhood and more specifically in rod and cone photoreceptor cells in adulthood and is seen to act as a cellular adhesive when functioning correctly. However, other proteins are also capable of fulfilling similar adhesive functions, for example adherins and cadherins in adherens junctions or claudins and occuldin in tight junctions. Given that some of these other proteins belong to large protein families, it is a possibility that variation in relative composition of these cellular adhesive protein cocktails may have a more notable contribution to a variable phenotype in the absence of functional retinoschisin.

This variability creates a diagnostic challenge in advanced XLRS1 disease. The clinical clues in conjunction with adjunctive testing, confirmed by robust genetic assessment (i.e. ruling out other causes and confirming a pathogenic *RS1* variant) confirm the diagnosis, thus empowering those affected by bringing them closer to accessing novel gene therapies where appropriate and preparing younger generations for those potential therapeutic options.

## Conclusions

Here we describe how multimodal imaging in XLRS1 and confirmation of a pathogenic *RS1* variant help to a) confirm diagnosis, b) monitor progression, c) assess clinical stage and thus suitability for novel therapeutic options even in patients of advanced age. Autofluorescence and OCT are non-invasive, well-tolerated and readily available in most clinical centres. Standardisation of clinical assessment and staging criteria, perhaps in conjunction with a central European reading centre, would allow detection of individuals with modifiable XLRS1 who may be suitable for novel gene therapy clinical trial participation.

## Abbreviations

ACMG: American College of Medical Genetics
AF: Autofluorescence
AO: Adaptive Optics
IRD: Inherited Retinal Degeneration
I-TASSER: Iterative Threading ASSEmbly Refinement
LogMAR: Logarithm of the Minimum Angle of Resolution
LOVD: Leiden University’s Open Variation Database
MMUH: Mater Misericordiae University Hospital, Dublin, Ireland
MPH: Mater Private Hospital, Dublin, Ireland
OCT: Optical Coherence Tomography
OMIM: Online Mendelian Inheritance in Man
PCR: Polymerase Chain Reaction
PDB: Protein Data Bank
RPE: Retinal Pigment Epithelium
RVEEH: Royal Victoria Eye & Ear Hospital, Dublin, Ireland
XLRS1: X-Linked Retinoschisis
